# Upper Lip Horizontal Line: Characteristics of a Dynamic Facial Line

**DOI:** 10.3390/ijerph17186672

**Published:** 2020-09-13

**Authors:** Alexander D Vardimon, Nir Shpack, Atalia Wasserstein, Marilena Skyllouriotou, Morris Strauss, Silvia Geron, Noa Sadan, Shifra Levartovsky, Rachel Sarig

**Affiliations:** 1 Department of Orthodontics, the Maurice and Gabriela Goldschleger School of Dental Medicine, Sackler Faculty of Medicine, Tel Aviv University, Tel Aviv 6997801, Israel; andyva@tauex.tau.ac.il (A.D.V.); nir@shpack.co.il (N.S.); ataliawa@gmail.com (A.W.); sky_marilena@yahoo.com (M.S.); mstrauss@barak.net.il (M.S.); sigeron@gmail.com (S.G.); sadan.noa@gmail.com (N.S.); 2 Department of Oral Rehabilitation, the Maurice and Gabriela Goldschleger School of Dental Medicine, Sackler Faculty of Medicine, Tel Aviv University, Tel Aviv 6997801, Israel; shifralevartov@gmail.com; 3 Department of Oral Biology, the Maurice and Gabriela Goldschleger School of Dental Medicine, Sackler Faculty of Medicine, Tel Aviv University, Tel Aviv 6997801, Israel; 4 The Dan David Center for Human Evolution and Biohistory Research, Sackler Faculty of Medicine, Tel-Aviv University, Tel Aviv 6997801, Israel

**Keywords:** upper lip, smile, sexual dimorphism, facial morphology, aging, wrinkle

## Abstract

*Background:* Upper lip appearance received major attention with the introduction of diverse treatment modalities, including lip augmentation, rhinoplasty surgery, and dental treatment designed to support the upper lip. Our objectives were to define the prevalence and characteristics of the upper lip horizontal line (ULHL), which is a dynamic line appearing during a smile, in relation to gender, malocclusions, aging, and facial morphology. *Methods:* First, the prevalence and gender distribution of ULHL was examined from standardized en-face imaging at full smile of 643 randomly selected patients. Second, cephalometric and dental cast model analyses were made for 97 consecutive patients divided into three age groups. *Results:* ULHL appears in 13.8% of the population examined, and prevailed significantly more in females (78%). The prevalence of ULHL was not related to age nor to malocclusion. Patients presenting ULHL showed shorter upper lip and deeper lip sulcus. The skeletal pattern showed longer mid-face, shorter lower facial height and greater prevalence of a gummy smile. *Conclusions:* Female patients with short upper lip, concavity of the upper lip, and gummy smile are more likely to exhibit ULHL. The ULHL is not age-related and can be identified in children and young adults. Therefore, it should be considered when selecting diverse treatment modalities involving the upper lip.

## 1. Introduction

Perceptions of facial beauty are multifactorial, with genetic, environmental, and cultural foundations [1]. Yet, various researchers who analyzed the esthetics of the smile and face revealed some common features of symmetry and harmony.

The face can be divided into thirds, with the lips comprising of the key aesthetic feature of the lower third, where the upper lip is shown as having the greatest effect on beauty perception. Traditionally, fuller lips have been considered more beautiful and were associated with voluptuousness, sensuality, and youthfulness. Indeed, in the last century, there has been a gradual increase in the demand for lip prominence [2], starting with dental treatment to support the upper lip through lip augmentation procedures and aesthetic plastic surgery [3,4,5,6]. Facial wrinkles that appear in the lower third of the face (i.e., nasolabial folds, vertical lip lines (perioral lines), Marionette lines (smile lines), and mental creases (chin lines)) might affect the attractiveness of the face. These wrinkles are affected by the orbicularis oris and 10 other circumferential muscles [7], as well as changes in lip and skin strain [8,9]. Vertical perioral lines in the upper lip develop with aging [10] and are more expressed in women [11].

The transverse upper labial crease was recently described to be located in the mid-philtral area below the nose [12]. It was visible both at rest and during animation or only during facial animation, and was more apparent in older women (>40). It was suggested that the anatomical basis for the formation of this crease is thought to be the insertion of the two depressor septi nasi muscle fibers into the overlying skin [13]. At least one third of the female volunteers were aware of this upper transverse labial crease, indicating that this might be of an aesthetic concern when attempting to address upper lip appearance [12].

Treatment of the upper lip region has received major attention lately, with the introduction of diverse modalities, such as injectable derivative of botulinum toxin [14], insertion of filler materials (e.g., hyaluronic acid) [15] or surgical techniques (e.g., myotomy of the Levator Labii Superioris Muscle [16], or lip lift and fat grafting) [17]. Although Botox has been primarily used in cosmetic treatment for lines and wrinkles on the face, it is also of great value and interest for dentists in an attempt to treat functional or aesthetic dental conditions, like deep nasolabial folds, radial lip lines, high lip line, and black triangles between teeth [18].

**Figure 1:**
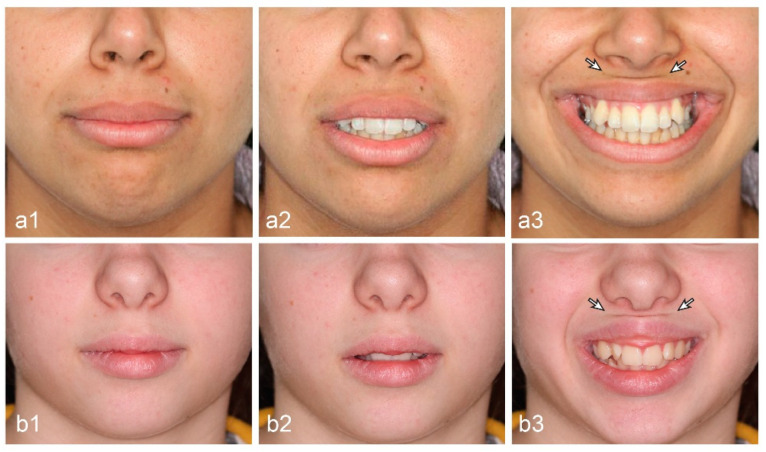
En-face view of two patients (**a**,**b**) with upper lip horizontal line (ULHL). (**a1**,**b1**): No presence of ULHL at lip together; (**a2**,**b2**): no presence of ULHL at rest; (**a3**,**b3**): full presence of ULHL at smile (see arrow). Note the flip of the upper lip during full smile.

In the present study, we describe the upper lip horizontal line (ULHL), which is a dynamic line that appears only while smiling (Figure 1). Although various parameters of the upper lip were analyzed in the past (e.g., lip length, naso-labial angle, gummy smile), no attention has been given yet to the ULHL and it has not yet been taken into diagnostic consideration.

When aesthetic treatment is planned, it is essential to match the expectations of the patient by having a complete diagnosis of all related parameters and to be aware of the clinician’s ability to affect the results. Even in cases in which the treatment plan does not include intervention (e.g., gummy smile, facial asymmetry), it is important to inform the patient.

Since ULHL is mainly visible during smiling (dynamic line), and in the light of diverse exposure of dental and gingival units during smiling [19], it is possible that ULHL is related to a predisposed spatial arrangement of soft and hard tissue in the face. Consequently, the question arises whether ULHL is a product of aging or a result of anatomical structure arrangement. In order to allow the clinician to achieve both functional and esthetical satisfaction of the patient, it is essential to reveal the possible association between the ULHL and other related features. Therefore, the objectives of the present study were to define the prevalence of ULHL in the population studied in relation to gender and malocclusions; to assess its persistence with aging and to examine the skeletal and soft tissue variables that are associated with its presence.

## 2. Materials and Methods

The study was approved by the Ethical Committee of the Tel Aviv University. Each patient or parent signed an informed consent form following the criteria of the Ethical Committee.

The study was divided into two stages. The first stage included a survey of a large sample to determine the prevalence of the ULHL in the population studied. The second stage aimed to evaluate the association of facial and dental features that were measured from cephalometric analyses (Figure 1) and study models. For the first stage, the sample consisted of 643 consecutively pretreated patients from the pool of patients treated at the Orthodontics Department in the School of Dental Medicine at Tel-Aviv University and at a private Orthodontic practice (Mo.St.). The sample was divided into three age groups (7–16, 17–26, 27–70y), with ages ranging from 7 to 70 years old and a mean age of 18.6 ± 9.18 years. This sample was used to examine the prevalence of the ULHL and its distribution in relation to patient gender and age.

The identification of the ULHL was based on standardized photographs of a full smile. Only patients who showed a clear fold in their upper lip during smile were categorized in the ULHL group. Malocclusion was analyzed based on the molar occlusion from dental study models following Angle classification—Class I molar occlusion, Class II molars with OJ greater than 2 mm (Class II/1), Class II molars with overjet (OJ) equal or smaller than 2 mm (Class II/2) and Class III. The presence or absence of a gummy smile was marked when exposure of more than 2 mm of gum above the upper central incisors was exposed while smiling [20]. The inclusion criteria were examined in pre-treatment en-face photos at rest and full smile. Subjects with facial deformities (e.g., cleft lip, right/left asymmetry), facial nerve paralysis (e.g., Bell’s palsy, hemifacial spasm), missing anterior teeth, and history of trauma or anterior restorations were excluded from the study. The second stage included 97 patients with pre-treatment lateral cephalometric radiographs and study models. All subjects with the ULHL (50 patients) and consecutively untreated patients without the ULHL that served as control (47 patients) were used for the second stage. The presence or absence of the ULHL was determined from standardized imaging of pretreatment en-face smiling (Figure 1), morphological features from lateral cephalometric radiographs, and study models.

**Figure 2:**
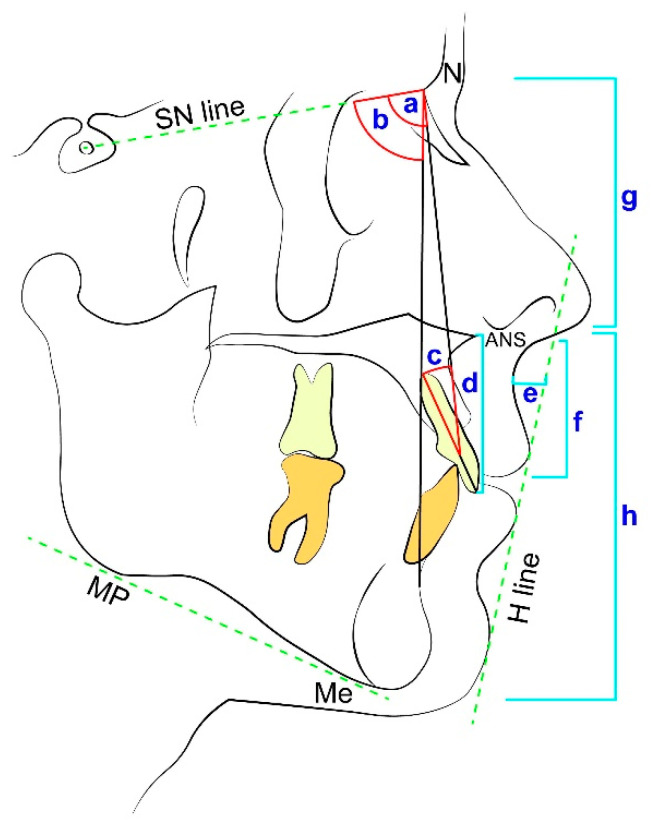
Cephalometric measurements used in the study: Sella–Nasion–A point angle (SNA) (a), Sella–Nasion–B point angle (SNB) (b), Upper incisors - Nasion- A point angle (U1 to NA) (c), incisor-maxilla height (d), sulcus depth (e) and upper lip height (f), vertical anterior upper proportions calculated by measured Nasion to anterior nasal spine (ANS) divided by Nasion-Menton (N-ANS/N-Me) (g) and vertical anterior lower proportions calculated by measured anterior nasal spine to Menton divided by Nasion-Menton (ANS-Me/N-Me) (h). Also marked in the figure: The Sella- Nasion line (SN line), the mandibular plane connecting point Gonion to Gnathion at the inferior border of the mandible (MP), Menton- the lowest point on mandibular symphysis (Me), the anterior nasal spine (ANS), Nasion- the most anterior point on frontonasal suture (N), and the H line, drawn tangent to the soft tissue chin and the upper lip.

Cephalometric analyses of nine skeletal and soft tissue measurements (Figure 2) were performed. These include: Sella to Nasion to A point angle (SNA), Sella to Nasion to B point angle (SNB), Upper incisor inclination to Nasion—A point line (U1-NA), Sella-Nasion line to mandibular plane (i.e., connecting point Gonion to Gnathion at the inferior border of the mandible) (SN-MP), vertical anterior upper proportion was calculated by measured Nasion to anterior nasal spine (ANS) divided by Nasion-Menton (N-ANS/N-Me) and vertical anterior lower proportion calculated by measured anterior nasal spine to Menton divided by Nasion-Menton (ANS-Me/N-Me), incisor-maxilla height (anterior nasal spine to incisal edge distance)—we use the term incisor-maxilla height to define the distance between the crown tip of the upper central incisor and ANS. This is due to a lack of accuracy in measuring the bone boundary at the incisor gingival margin on lateral cephalogram radiographs—upper lip height (the horizontal distance between subnasale and stomion superior), and sulcus depth (the horizontal distance measured from the deepest point of the upper lip curvature to H-line) (Figure 2). The statistical analysis was carried out using SPSS software for Windows (version 20; SPSS, Chicago, IL, USA). The reliability of the measurements was evaluated on 15 patients, using the Intra Class Correlation Coefficient (ICC) measured twice by the same examiner, ten days apart. All nine cephalometric parameters were evaluated. Kolmogorov–Smirnov tests were carried out to verify the normality of the measurement distributions. The Chi-square test was used for the prevalence analysis; the T-test was used to compare numerical values of skeletal and soft tissue variables between subjects with and without ULHL. The significance level was set at *p* < 0.05. To predict the presence of the ULHL, a stepwise logistic regression test was used.

## 3. Results

The reliability Intra Class Correlation Coefficient (ICC) presented high correlations in all cephalometric measurements taken (ICC > 0.9).

### 3.1. ULHL—Prevalence by Gender

Out of the total sample (N = 643), the prevalence of the ULHL was 13.8% (N = 89). The ULHL was significantly more prevalent in females (based on Chi-square *p* < 0.001): 83% females (N = 74) and 17% males (N = 15) (Table 1).

### 3.2. ULHL—Prevalence by Age

There was no significant difference in the average age between the group presenting the ULHL (17.78 ± 9.54 years) and the group without this feature (18.72 ± 9.06 years) (based on T–Test).

**Figure 3:**
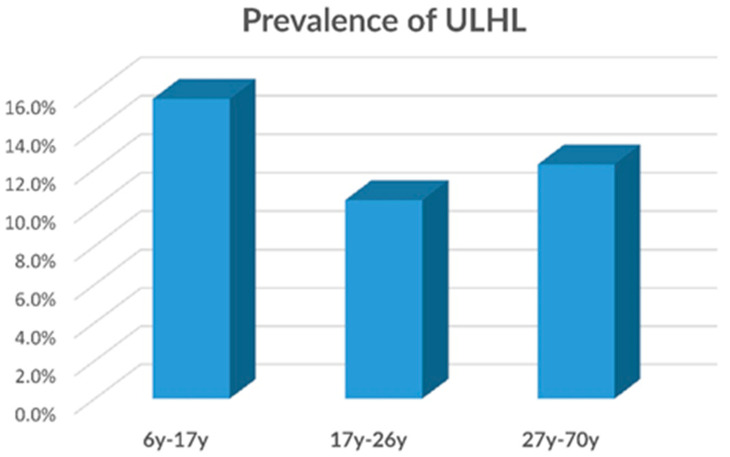
Prevalence of the ULHL according to three age groups, based on the first group survey (n = 643). No significant difference was found between the three age groups.

No significant difference was found in the prevalence of the ULHL between the three age groups (i.e., 7–16, 17–26, 27–70y) (based on the Pearson Chi-square analysis) (Figure 3). The female sample presented gradual decrease (although not significantly) with age in ULHL prevalence, ranging from 14.3% in the young age group to 10.9% and 9.5% in the older age groups.

### 3.3. ULHL—Prevalence by Malocclusions

No significant difference was found between different malocclusions and the prevalence of ULHL (Table 2). Most ULHL cases had Class I and Class II/div. 1 malocclusions, similar to the prevalence in the group without ULHL (no-ULHL).

### 3.4. ULHL—Prevalence by Gummy Smile

Significantly greater prevalence of ULHL was found in patients presenting a gummy smile (27.8%) as compared to patients without a gummy smile (15.2%) (*p* = 0.004) (Table 3).

### 3.5. ULHL—Cephalometric Measurements

Out of the nine cephalometric measurements, four measurements were significantly different in the ULHL group in both males and females: the upper lip was significantly shorter in patients presenting ULHL (*p* < 0.028), as well as greater sulcus depth was found in the ULHL group (*p* < 0.017), greater upper anterior vertical proportion (N-ANS/N-Me) (*p* < 0.028), and smaller lower anterior vertical proportion (ANS-Me/N-Me) (*p* < 0.026) (Table 4).

### 3.6. ULHL—Linear Regression

A stepwise logistic regression was used to evaluate which of the parameters can best predict the presence of ULHL (R = 0.693, R square = 0.48, *p* < 0.001). The parameters that were included in the analysis were: the upper lip height, sulcus depth, and gender (Table 5).

## 4. Discussion

The most common criterion for the patient in order to assess the successfulness of the treatment is smile attractiveness [21], therefore achieving a balanced smile is an important goal for the clinician [22]. Intraorally, dental smile parameters were thoroughly described (smile width, buccal corridor, smile arc, occlusal cant, lateral step, central incisor width-to-length ratio, canine to lateral incisor ratio) [22]. Extra orally, one of the major criteria in restoring the upper anterior dentition is the position of the upper lip during smile in relation to the incisal edges [23,24,25,26,27]. This relationship deviates from the norm posture in case of short upper lip or long upper lip with respect to gender [28,29] and age [28,30,31]. Short upper lip often leads to the appearance of a gummy smile [8], an overexposure of the labial maxillary gingiva during smile (>2–3 mm) [9]. Long upper lip is often related to the ascending of the upper lip with aging, causing upper incisor coverage during smile [30]. However, although the effect of upper lip position on smile was thoroughly studied, the effect of adjacent wrinkles on smile and tooth exposure was barely addressed. Moreover, differential facial preferences are gender, ethnicity, and age-specific and therefore might affect patients’ satisfaction [32,33].

In the present study, we described and analyzed for the first time morphological features of the upper lip horizontal line, which we defined as the ULHL. Furthermore, we raised the question of whether the presence of ULHL during smile is related to aging, similar to the labial horizontal crease, or if it relates to a distinct spatial arrangement of soft and hard tissues in the face. That is, which of the two etiologies dictates the development of a horizontal folding of the upper lip during smile.

When comparing our findings with those of Beer and Manestar [12], although some similarity can be noted, it seems that the ULHL and the transverse crease present different features; the ULHL appears in smile with the line extending laterally beyond the philtrum, whereas the transverse crease is confined to the mid-philtral area. The ULHL is not age-related, whereas the transverse crease is age-related and appears primarily in women over 40 years of age [12].

If ULHL was age-related (similar to facial wrinkles or labial horizontal crease), we would expect an increase in ULHL presence with aging. Surprisingly, this was not the case. No statistical difference was found between the age groups. On the contrary, there was a tendency of ULHL prevalence to decline with age. The almost plateau level of ULHL prevalence that remained between all age groups suggests that subjects who have ULHL at an early age will still display it at an elderly age. The slight decrease in ULHL prevalence with aging can be explained by the drop of the upper lip [10,34,35] and muscle fatigue [36]. This can be explained due to the fact that maximal horizontal smile decreases with aging (decrease in activity of m. Levator Labii Superioris, m. Zygomaticus Minor, m. Zygomaticus Major, and m. Risorius), resulting in a lesser decrease in upper lip height with no folding of the lip and a non-appearance of ULHL during smile. While we could not show the decline of ULHL presence with age statistically, that change over time is most likely due to a shortage of patients in the third age group.

Hwang et al. [14] defined three muscles involved in the upper lip elevation, i.e., Levator Labii Superioris, Levator Labii Superioris Alaeque Nasi, and Zygomaticus Minor. With relation to these muscles, the injection points for applying botulinum toxin in order to decrease upper lip muscle activity were defined as where the three muscles overlap, which consequently eliminates gummy smile. We assume that muscle hyperactivity (Levator Labii Superioris, Levator Labii Superioris Alaeque Nasi, and Zygomaticus Minor), combined with the three traits defined in the present study (sexual dimorphism, short upper lip, and sulcus depth), are predisposed factors for ULHL emergence during smile. The presence or hyperactivity of the depressor septi nasi muscle has been proposed to cause a visible transverse crease in the upper labial region that is aesthetically disturbing to patients [12]. However, it is plausible that hyperactivity of additional muscles involved in the formation of the ULHL, including Levator Labii Superioris and Levator Labii Superioris alaeque nasi that are oriented almost vertically [37], might affect the pull and flip of the upper lip.

Alongside the hyperactivity of the mimic muscles, it was suggested that the superficial musculoaponeurotic system (SMAS) around the nasolabial fold, with its condensing fibro-muscular meshwork, is responsible for the nasolabial fold formation and other facial creases/lines [38,39]. The finding that ULHL is not age-related suggests that these features might be genetically induced, therefore further studies are required.

ULHL was found to be related to long mid-face (N-ANS/N-Me), short maxilla (height of maxilla), short lower face (ANS-Me/N-Me), short upper lip (upper lip height), and concavity of the upper lip (sulcus depth). The ULHL was more prevalent in females, which is supported by studies related to gender dimorphism in the lip region and unique characteristics to the female smile line [40,41,42], as well as by the study of Miron et al. [43], who discovered that short upper lip is more common in females.

Improvement of the upper lip aesthetic should be considered as part of dental treatment plan. There are several approaches to correct a gummy smile or soft tissue asymmetry, such as administration of Botulinum toxin or rhinoplasty procedures [18]. However, no study examined the effect of treatment modalities on the ULHL. We can only speculate that the most popular rhinoplasty procedures of the resection of the depressor septi nasi muscle might be inadequate for complete ULHL elimination, therefore a preliminary examination with Botulinum toxin might give an indication for possible treatments.

The clinician should take into consideration that the presence of the ULHL can affect the satisfaction of the patient, similarly to the presence of a gummy smile or smile asymmetry. Therefore, we recommend including the diagnosis of the ULHL in smile analysis and, when needed, further intervention should be recommended.

## 5. Conclusions

In the present study, we report on the presence of an upper lip horizontal line (ULHL), which appears in 13.8% of the sample while smiling. This line can be associated with morphological features, such as short upper lip and concavity of the lip in the philtrum region. The ULHL is found in a higher prevalence in females compared to males. It is not related to malocclusion, nor to aging.

Patients exhibiting ULHL should be informed prior to dental treatment that the ULHL is a morphological feature and is not related to aging, and clinicians should be aware of the possible treatment modalities that can be presented to the patient to improve smile esthetics.

## Figures and Tables

**Table 1:** Descriptive analysis of the study samples: the survey group (First stage) and the analyzed group (Second stage).

			Individuals Presenting with ULHL			
		All Group	Prevalence		Age	
		N	N	%	Mean	Std
**First stage group**	**Males**	235	15	6.4	17.12	8.149
	**Females**	408	74	18.1	19.39	9.580
	**Total**	643	89	14.0	18.60	9.190
**Second stage group**	**Males**	34	9	26.5	14.93	4.764
	**Females**	63	41	65.1	17.63	8.678
	**Total**	97	50	51.5	16.68	7.623

**Table 2:** Chi-square table presenting the prevalence of the ULHL and without it (no-ULHL), according to malocclusion. No significant difference was found in the prevalence of the ULHL between the different malocclusions.

		Malocclusion				Total
		Cl I	Cl II/1	Cl II/2	Cl III	
**ULHL**	**N**	48	31	4	7	90
	**%**	53.3%	34.4%	4.4%	7.8%	100.0%
**no-ULHL**	**N**	224	234	36	59	553
	**%**	40.5%	42.3%	6.5%	10.7%	100.0%

**Table 3:** The prevalence of the ULHL and without it (no-ULHL) according to a gummy smile. Based on the Chi-square analysis, a significant difference (*p* = 0.004) was found in the prevalence of ULHL in the presence of a gummy smile.

		Gummy Smile	Non-Gummy Smile	Total
**ULHL**	**N**	25	65	90
	**%**	27.8%	72.2%	100.0%
**no-ULHL**	**N**	84	469	553
	**%**	15.2%	84.8%	100.0%
**Total**	**N**	109	534	643
	**%**	17.0%	83.0%	100.0%

**Table 4:** Cephalometric measurements of the study group with and without ULHL, T-test analysis was used to describe the differences between the two groups.

Cephalometric Measurement	ULHL		No ULHL		*p* Value	95% Confidence Interval of the Difference	
	MEAN	STD	MEAN	STD		Lower	Upper
**SNA (°)**	81.84	4.11	81.26	4.18	NS	−1.09	2.26
**SNB (°)**	78.14	3.51	78.65	3.69	NS	−1.96	0.94
**U1 to NA (°)**	26.86	7.40	25.37	9.52	NS	−1.94	4.91
**SN to MP (°)**	33.95	5.61	33.51	7.01	NS	−2.11	2.99
**N-ANS/N-Me (%)**	44.62	3.08	42.40	3.15	0.001	0.96	3.47
**ANS-Me/N-Me (%)**	55.36	3.08	57.64	3.12	>0.001	−3.53	−1.03
**Incisor-maxilla height (mm)**	26.00	3.97	27.98	3.26	0.009	−3.45	−0.51
**Upper lip height (mm)**	19.42	2.45	23.06	3.05	>0.001	−4.76	−2.53
**Sulcus depth (mm)**	6.54	2.21	5.40	1.93	0.008	0.30	1.97

**Table 5:** Stepwise linear logistic regression analysis to predict the ULHL presence.

Model		95% Confidence Interval for B	
	Beta	Lower	Upper
**Upper lip height (mm)**	0.535	0.057	0.107
**Sulcus depth (mm)**	−0.394	−0.128	−0.057
**Gender**	−0.213	−0.393	−0.054

## List of Abbreviations

Sella	Midpoint of sella turcica
A point	Most concave point of anterior maxilla
B point	Most concave point on mandibular symphysis
Menton	Lowest point on mandibular symphysis
Nasion	Most anterior point on frontonasal suture
ANS	The anterior nasal spine
SNA (°)	Sella-Nasion-A point angle
SNB (°)	Sella-Nasion-B point angle
U1 to NA (°)	Upper incisors–Nasion-A point angle
SN to MP (°)	Sella- Nasion to mandibular plane angle
N-ANS/N-Me	Nasion-Anterior nasal spine/Nasion-Menton
ANS-Me/N-Me	Anterior nasal spine-Menton/Naion-Menton
Mandibular plane (MP)	The plane connecting Gonion to Gnathion at the inferior border of the mandible
H line	Drawn tangent to the soft tissue chin and the upper lip
